# Supplementary material for: Symmetry-protected solitons and bulk-boundary correspondence in generalized Jackiw–Rebbi models
Source: Sci Rep. 2021 Nov 4;11:21652. doi: 10.1038/s41598-021-01117-5 (PMC8569174; doi:10.1038/s41598-021-01117-5)
Supplement: Supplementary file 1 — Supplementary Information. [file 41598_2021_1117_MOESM1_ESM.pdf]

Supplemental Information: Symmetry-Protected Solitons and Bulk-Boundary Correspondence in Generalized Jackiw-Rebbi Models

Chang-geun Oh,[1] Sang-Hoon Han,[2] and Sangmo Cheon[1,2,3]

[1] *Research Institute for Natural Sciences, Hanyang University, Seoul 04763, Korea*
[2] *Department of Physics, Hanyang University, Seoul 04763, Korea*
[3] *Institute for High Pressure, Hanyang University, Seoul, 04763, Korea*

CONTENTS

S1. Jackiw-Rebbi model 2
S1.1. Model 2
S1.2. Energy spectrum 2
S1.3. $\mathcal{T}, \mathcal{C}, \mathcal{P}$ symmetries 2
S1.4. Duality of JR solitons 2

S2. Generalized Jackiw-Rebbi model 3
S2.1. Model 3
S2.2. Energy spectrum 3
S2.2.1. Bulk spectrum 3
S2.2.2. Soliton modes 4
S2.2.3. Lorentz violation terms 4
S2.3. Fermion number 5
S2.4. Comparison with the tight-binding model 6
S2.5. $\mathcal{T}, \mathcal{C}, \mathcal{P}$ symmetries and dualities among solitons 6
S2.5.1. Bulk and NC soliton systems 6
S2.5.2. RC and LC soliton systems 6
S2.6. Field rotation symmetry 7

References 7

S1. JACKIW-REBBI MODEL

S1.1. Model

The Lagrangian density of the Jackiw-Rebbi (JR) model [1, 2] with a complex fermion mass is given by

$$\mathcal{L}_{\text{JR}} = \bar{\psi}[i\gamma^0\partial_0 + i\gamma^1\partial_1 - \phi(x) - i\gamma^5 m_z]\psi, \tag{1}$$

where $\gamma^0 = \sigma^y, \gamma^1 = -i\sigma^z, \gamma^5 = \sigma^x$, m_z is a fermion mass, and $\phi(x)$ is a bose field in the double-well potential $V(\phi)$ that has two degenerate minima at $\phi = \pm\phi_0$. When $m_z = 0$, the Lagrangian density describes the low-energy effective theory of the Su-Schrieffer-Heeger (SSH) model [1–3]. When $m_z \neq 0$, the Lagrangian density describes the Rice-Mele (RM) model [4].

S1.2. Energy spectrum

The energy spectrum can be obtained by the one-particle Hamiltonian. For the homogeneous bose field in a minimum $\phi(x) = \phi_0$, the gapped energy spectrum of a JR model is given by $E = \pm\sqrt{k^2 + \phi_0^2 + m_z^2}$.

On the other hand, when the bose field is a soliton field [$\phi(x) = \phi_s(x)$] that varies spatially and connects the two degenerate minima [$\phi_s(\pm\infty) = \pm\phi_0$], there appears the isolated energy mode $E_s = m_z$ in the gap. For an anti-soliton field [$\phi(x) = \phi_{\bar{s}}(x) = -\phi_s(x)$], an isolated energy mode appears at $E_s = -m_z$. For the SSH model, all isolated energy mode are zero modes.

S1.3. $\mathcal{T}, \mathcal{C}, \mathcal{P}$ symmetries

For the SSH model, the Lagrangian density in Eq. (1) has $\mathcal{T}$, $\mathcal{C}$, $\mathcal{P}$ symmetries:
(i) Time reversal $t \to -t$

$$\mathcal{T}\psi(t,x)\mathcal{T}^{-1} = \gamma^5\gamma^0\psi(-t,x) \tag{2}$$

(ii) Charge conjugation

$$\mathcal{C}\psi(t,x)\mathcal{C}^{-1} = \psi^*(t,x) \tag{3}$$

(iii) Parity $x \to -x$

$$\mathcal{P}\psi(t,x)\mathcal{P}^{-1} = \gamma^0\psi(t,-x) \tag{4}$$

When the bose field is a soliton field, an additional parity transformation for the bose field $\mathcal{P}\phi_s(x)\mathcal{P}^{-1} = \phi_s(-x)$ should be accompanied. Therefore, the soliton system composed of $\psi_s(x)$ and $\phi_s(x)$ is self-charge conjugate [2].

For the RM model, $\mathcal{C}$ and $\mathcal{P}$ symmetries are broken due to the fermion mass m_z, while $\mathcal{T}$ symmetry is preserved.

S1.4. Duality of JR solitons

We prove that a soliton system composed of $\psi_s(x)$ and $\phi_s(x)$ and the antisoliton system composed of $\psi_{\bar{s}}(x)$ and $\phi_{\bar{s}}(x)$ in the JR model form a charge conjugation and parity pair. That is, the charge conjugated field ($\psi_s^{\mathcal{C}}$) for a soliton system can be described with the help of the field ($\psi_{\bar{s}}$) in the antisoliton system and vice versa:

$$\psi_s^{\mathcal{C}} = \mathcal{C}\psi_s\mathcal{C}^{-1} = -\gamma^5\psi_{\bar{s}}^* \tag{5}$$

This can be proved by the equation of motion.

$$\text{e.o.m for soliton system:} \quad [i\gamma^0(\partial_0 - ieA_0) + i\gamma^1(\partial_1 - ieA_1) - \phi_s - i\gamma^5 m_z]\psi_s = 0, \tag{6}$$

$$\text{e.o.m for charge conjugated soliton system:} \quad [i\gamma^0(\partial_0 + ieA_0) + i\gamma^1(\partial_1 + ieA_1) - \phi_s - i\gamma^5 m_z]\psi_s^{\mathcal{C}} = 0, \tag{7}$$

$$\text{e.o.m for antisoliton system:} \quad [i\gamma^0(\partial_0 - ieA_0) + i\gamma^1(\partial_1 - ieA_1) + \phi_s - i\gamma^5 m_z]\psi_{\bar{s}} = 0, \tag{8}$$

$$[i\gamma^0(\partial_0 + ieA_0) + i\gamma^1(\partial_1 + ieA_1) - \phi_s - i\gamma^5 m_z](-\gamma^5)\psi_{\bar{s}}^* = 0. \tag{9}$$

From Eq. (8), one can obtain Eq. (9). Eq. (9) shows that $-\gamma^5\psi_{\bar{s}}^*$ satisfies the same equation of motion for charge conjugated soliton system in Eq. (7), which confirms Eq. (5).

Similarly, the parity transformed field for a soliton system can be described by the help of the field in an antisoliton system, and vice versa:

$$\mathcal{P}\psi_s(t,x)\mathcal{P}^{-1} = \gamma^0\gamma^5\psi_{\bar{s}}(t,-x), \tag{10}$$

$$\mathcal{P}\phi_s(x)\mathcal{P}^{-1} = \phi_s(-x) = \phi_{\bar{s}}(x). \tag{11}$$

S2. GENERALIZED JACKIW-REBBI MODEL

S2.1. Model

The Lagrangian density of the generalized JR model is

$$\mathcal{L} = \mathcal{L}_1 + \mathcal{L}_2 + \mathcal{L}_{\text{int}} + \mathcal{L}_{\text{B}}, \tag{12}$$

$$\mathcal{L}_j = \bar{\Psi}_j[i\partial\!\!\!/ - \Phi_j]\Psi_j, \tag{13}$$

$$\mathcal{L}_{\text{int}} = -\bar{\Psi}_1[t_1\gamma^0 - it_2\gamma^1]\Psi_2 - \bar{\Psi}_2[t_1\gamma^0 + it_2\gamma^1]\Psi_1, \tag{14}$$

$$\mathcal{L}_{\text{B}} = \frac{1}{2}(\partial_\mu\Phi_1\partial^\mu\Phi_1 + \partial_\mu\Phi_2\partial^\mu\Phi_2) - \frac{K}{2}(\Phi_1^2 + \Phi_2^2), \tag{15}$$

where Ψ_j and Φ_j are two-component spinor and real scalar bose fields ($j = 1, 2$). In this model, we minimally coupled two JR models by adding the inter-field coupling between Dirac fermion fields. Therefore, the potential for the Bose fields $U = -\frac{K}{2}(\Phi_1^2 + \Phi_2^2)$ has a $O(2)$ rotational symmetry. However, the total energy potential has a discrete symmetry (Z_2 or Z_4) depending on the interfield couplings, as discussed in the main text. For simplicity, we do not consider the possible interactions between bosons because we focus on the fermion fields and treat the bose fields as static background fields.

Using Clifford matrices, the Lagrangian density in Eq. (12) can be rewritten as follows:

$$\mathcal{L} = \bar{\mathbf{\Psi}}[\Gamma_2 i\partial_0 + \Gamma_3\partial_1 + t_1\Gamma_{34} - it_2\Gamma_{25} - \mathbf{\Phi}]\mathbf{\Psi} + \mathcal{L}_{\text{B}}, \tag{16}$$

where $\mathbf{\Psi} = (\Psi_1, \Psi_2)$, $\mathbf{\Phi} = \begin{pmatrix} \Phi_1 & 0 \\ 0 & \Phi_2 \end{pmatrix}$, $\bar{\mathbf{\Psi}} = \mathbf{\Psi}^\dagger\Gamma_2$. Here, Γ_a and Γ_{ab} ($a, b = 1, \cdots 5$) are gamma matrices. They are chosen as $\Gamma_a = (\sigma_z \otimes \tau_x, 1 \otimes \tau_y, 1 \otimes \tau_z, \sigma_x \otimes \tau_x, \sigma_y \otimes \tau_x)$, and $\Gamma_{ab} = \frac{1}{2i}[\Gamma_a, \Gamma_b]$.

S2.2. Energy spectrum

S2.2.1. Bulk spectrum

Here, we calculate the energy spectrum in a global minimum. From Eq. (16), we obtain 4×4 one-particle Hamiltonian. When the bose fields are in A or C minimum in Fig. 4(c), for instance, the one-particle Hamiltonian can be written as

$$\mathsf{H} = \sigma_0 \otimes [k_x\tau_x \pm \Phi_0\tau_y] + [t_1\sigma_x \otimes \tau_0 + t_2\sigma_y \otimes \tau_x], \tag{17}$$

where $+\Phi_0$ ($-\Phi_0$) is for A (C) minimum. The energy spectrum is given by which gives the following energy spectrum.

$$E = \pm\sqrt{\frac{A(\Phi_0, \Phi_0) \pm B(\Phi_0, \Phi_0)}{2}}, \tag{18}$$

$$A(\Phi_1, \Phi_2) = 2(k_x^2 + t_1^2 + t_2^2) + (\Phi_1^2 + \Phi_2^2), \tag{19}$$

$$B(\Phi_1, \Phi_2) = \sqrt{16(t_1^2 + t_2^2)k_x^2 + 4(t_1^2 + t_2^2)(\Phi_1^2 + \Phi_2^2) + 8(t_1^2 - t_2^2)\Phi_1\Phi_2 + (\Phi_1^2 - \Phi_2^2)^2}. \tag{20}$$

From this energy spectrum, we quickly check that the bands are degenerated when $t_1 = t_2 = 0$, while the bands are split when $t_1 \neq 0$ or $t_2 \neq 0$. Similarly, for the B and D minima, the same energy spectrum can be obtained except for the exchange between t_1 and t_2.

The energy spectrum can also be calculated by using 2×2 effective one-particle Hamiltonian for each JR fields. From Eq. (12), we get the following equations of motion.

$$\left[i\partial\!\!\!/ - \Phi_1\right]\Psi_1 = \left[t_1\gamma^0 - it_2\gamma^1\right]\Psi_2, \qquad \left[i\partial\!\!\!/ - \Phi_2\right]\Psi_2 = \left[t_1\gamma^0 + it_2\gamma^1\right]\Psi_1. \tag{21}$$

By inserting Eq. (21) into Eq. (12), the Lagrangian density of fermions can be described as two effective Lagrangian densites

$$\mathcal{L} = \mathcal{L}_1 + \mathcal{L}_2 + \mathcal{L}_{\text{int}} = \mathcal{L}_1^{\text{eff}} + \mathcal{L}_2^{\text{eff}}, \tag{22}$$

$$\mathcal{L}_j^{\text{eff}} = \bar{\Psi}_j[i\partial\!\!\!/ - \Phi_j]\Psi_j - \bar{\Psi}_j[t_1\gamma^0 - (-1)^{j-1}it_2\gamma^1]\frac{1}{i\partial\!\!\!/ - \Phi_j}[t_1\gamma^0 + (-1)^{j-1}it_2\gamma^1]\Psi_j. \tag{23}$$

For a constant or slowly-varying soliton bose field, the gradient of Φ_j is zero or very small. Then, the effective Lagrangian density in Eq. (23) can be approximated as

$$\mathcal{L}_j^{\text{eff}} \approx \bar{\Psi}_j[i\partial\!\!\!/ - \Phi_j]\Psi_j + \bar{\Psi}_j\left[t_1^2\frac{i\gamma^0\partial_0 - i\gamma^1\partial_1 + \Phi_{3-j}}{\Box + \Phi_{3-j}^2} + t_2^2\frac{i\gamma^0\partial_0 - i\gamma^1\partial_1 - \Phi_{3-j}}{\Box + \Phi_{3-j}^2} + (-1)^{j-1}2t_1t_2\frac{i\gamma^5\Phi_{3-j}}{\Box + \Phi_{3-j}^2}\right]\Psi_j. \tag{24}$$

From each effective Lagrangian density in Eq. (24), the following effective one-particle Hamiltonian H_j for the positive frequency is obtained.

$$\mathsf{H}_j = \left[-i\partial_x\sigma_x + \Phi_j\sigma_y + \frac{t_1^2+t_2^2}{E^2+\partial_x{}^2-\Phi_{3-j}^2}(E-i\partial_x\sigma_x) + \frac{(t_1^2-t_2^2)\Phi_{3-j}}{E^2+\partial_x{}^2-\Phi_{3-j}^2}\sigma_y + \frac{(-1)^{j-1}2t_1t_2\Phi_{3-j}}{E^2+\partial_x{}^2-\Phi_{3-j}^2}\sigma_z \right]. \quad (25)$$

By considering a plane-wave solution, the energy spectrum is given by $E=\pm\sqrt{\frac{A(\phi_1,\phi_2)\pm B(\phi_1,\phi_2)}{2}}$, which gives the same results in Eq. (18).

S2.2.2. Soliton modes

The isolated energy spectrum of the fermion field in the background soliton bose fields can be easily obtained with the effective Hamiltonians.

As an example, we consider the RC soliton system of which bose fields interpolate from A to B minima as shown in Fig. 2(d), $\Phi_1(\pm\infty)=\Phi_s(\pm\infty)=\mp\Phi_0$ and $\Phi_2(x)=\Phi_0$. Since the RC soliton can exist only at the critical point $t_1=t_2$, the effective one-particle Hamiltonian in Eq. (25) becomes

$$\mathsf{H}_j = \left[-i\partial_x\sigma_x + \Phi_j\sigma_y + \frac{2t_1^2}{E^2+\partial_x{}^2-\Phi_{3-j}^2}(E-i\partial_x\sigma_x) + \frac{(-1)^{j-1}2t_1^2\Phi_{3-j}}{E^2+\partial_x{}^2-\Phi_{3-j}^2}\sigma_z \right]. \quad (26)$$

As discussed in the main text, there are two isolated soliton modes. For an RC soliton system, the primary mode appears mainly in (Ψ_1,Φ_1) while the induced mode does in (Ψ_2,Φ_2). Thus, H_1 and H_2 can be used to calculate the energy spectra of primary and induced modes, respectively.

We first calculate the spectrum of the primary mode. When $|x|\gg 1$, from Eq. (26), the energy eigenvalue equation for the primary mode can be approximated as

$$\left[-i\partial_x\sigma_x + \frac{E^2-\Phi_0^2}{E^2-\Phi_0^2+2t_1^2}\Phi_s(x)\sigma_y + \frac{2t_1^2}{E^2-\Phi_0^2+2t_1^2}\Phi_0\sigma_z \right]\psi_{\mathrm{P}} \approx E\frac{E^2-\Phi_0^2-2t_1^2}{E^2-\Phi_0^2+2t_1^2}\psi_{\mathrm{P}}, \quad (27)$$

because the amplitude of the isolated soliton mode vanishes as it moves away from the soliton center.

By solving Eq.(27), the energy spectrum and wavefunction of the primary mode are given by

$$E_{\mathrm{P}} = -\frac{2t_1^2}{\Phi_0} + O(t_1^4), \quad \psi_{\mathrm{P}} \approx \begin{pmatrix} \exp^{\left[\int^x dx'\Delta_s(x')\right]} \\ 0 \end{pmatrix}, \quad (28)$$

where $\Delta_s(x)=\frac{E_P^2-\Phi_0^2}{E_P^2-\Phi_0^2+2t_1^2}\Phi_s(x)$. Similarly, the energy spectrum of the induced mode can be obtained as $E_I=-\Phi_0+t_1-\frac{t_1^2}{4\Phi_0}+O(t_1^3)$ using H_2, where one can also use a proper unitary transformation of $\psi_I\to\psi_I'=\frac{1}{\sqrt{2}}(1-i\sigma_x)\psi_I$ for convenience. In the same way, the energy spectra of soliton modes in LC soliton systems can be obtained.

For NC soliton systems, the energy spectra of the hybridized bonding and antibonding soliton modes can also be calculated regardless of t_1 and t_1 in a similar way. For a more straightforward calculation, one can use the hybridized bonding and antibonding fields. For instance, $\Psi_{\mathrm{B}}=\frac{1}{\sqrt{2}}[\Psi_1-\Psi_2]$ and $\Psi_{\mathrm{AB}}=\frac{1}{\sqrt{2}}[\Psi_1+\Psi_2]$ can be used in the NC soliton system of which bose fields interpolate from A to C minima. The calculated spectra are plotted in Fig. 3 for $t_1=t_2$ case and in Fig. S1 for $t_1\neq t_2$ case.

S2.2.3. Lorentz violation terms

Here, we discuss the Lorentz violating terms in the effective Lagrangian density and their effects on the spectra of the soliton modes. When $t_1=t_2$, from Eq. (24), the effective Lagrangian density for the jth field is given by

$$\mathcal{L}_j^{\mathrm{eff}} = \bar{\Psi}_j[i\partial\!\!\!/ - \Phi_j]\Psi_j + \bar{\Psi}_j\left[2t_1^2\frac{i\gamma^0\partial_0 - i\gamma^1\partial_1 + (-1)^{j-1}i\gamma^5\Phi_{3-j}}{\Box+\Phi_{3-j}^2}\right]\Psi_j. \quad (29)$$

This effective Lagrangian has two types of Lorentz violating terms: A chiral mass term which is proportional to $2t_1^2\Phi_{3-j}\gamma^5$ and two space-time deformation terms which are proportional to $2t_1^2\gamma^1$ and $2t_1^2\gamma^0$, respectively. Even though both can give corrections to the physical quantities, the space-time deformation terms give higher-order corrections and hence can be ignored in the leading order. For example, consider the energy spectrum of the primary mode in an RC soliton system. If we calculate the energy spectrum ignoring the space-time deformation terms, we have $E_{\mathrm{P}}=-\frac{2t_1^2}{\Phi_0}+O(t_1^4)$, which gives the same result with Eq. (28) up to t_1^3 order.

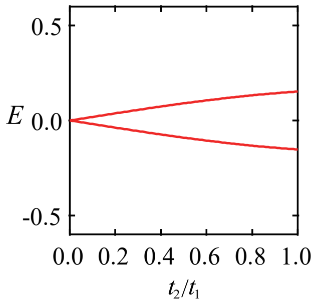

Fig. S1. Energy spectra for bonding and antibonding modes in an NC soliton system with respect to t_2/t_1. The upper and lower lines represent the energy spectra for antibonding and bonding modes, respectively. The spectra are normalized by the energy gap. Here, $t_1 = 0.1$. When $t_2/t_1 = 0$, bonding and antibonding become two degenerate zero modes even in the presence of the interfield coupling, which is consistent with the ladder-SSH model [5].

S2.3. Fermion number

The zero mode in the SSH model has the quantized $\frac{1}{2}$ fermion number [1, 3]. Similarly, the isolated soliton modes in the generalized JR model have fractional fermion numbers. We calculate the fermion number of each isolated soliton mode using the Goldstone-Wilczek method [6] and effective Lagrangian densities.

As an example, we calculate the fractional fermion number of the isolated modes in the RC soliton system of which bose fields interpolate from A to B minima, i.e, $\Phi_1(\pm\infty) = \Phi_s(\pm\infty) = \mp\Phi_0$ and $\Phi_2(x) = \Phi_0$. First, we consider the fermion number of the primary mode, which mainly exists in Ψ_1. From Eq. (29), the effective Lagrangian density for Ψ_1 can be approximated as

$$\mathcal{L}_1^{\text{eff}} \approx \bar{\Psi}_1[i\partial\!\!\!/ - (\Phi_s - i\gamma^5 \frac{2t_1^2\Phi_0}{\Phi_0^2 - E^2})]\Psi_1. \tag{30}$$

Here, up to leading order, we ignore space-time deformations because they give the higher order correction similar to the energy spectrum as we discussed in S2.2.3.

Note that E^2 in Eq. (30) has two degrees of freedom because there are two occupied bands in the energy spectrum in Eq. (18): $E_+^2 = \frac{A(\Phi_1,\Phi_2)+B(\Phi_1,\Phi_2)}{2}$, $E_-^2 = \frac{A(\Phi_1,\Phi_2)-B(\Phi_1,\Phi_2)}{2}$. Then, the induced current $\left\langle j^\mu_{E_\pm}(x)\right\rangle$ and the fermion number $Q(E_\pm)$ for each bands $E_\pm$ are given by

$$\left\langle j^\mu_{E_\pm}(x)\right\rangle = -\frac{1}{2\pi}\epsilon^{\mu\nu}\partial_\nu\theta, \tag{31}$$

$$Q(E_\pm) = \int \text{dx}\; j^\mu_{E_\pm}(x), \tag{32}$$

where

$$\tan\theta \equiv -\frac{2t_1^2\Phi_0}{(\Phi_0^2 - E_\pm^2)\Phi_s(x)}. \tag{33}$$

Hence, the fermion number of the primary mode is given by

$$Q_{\text{P}}^{\text{RC}} = \frac{1}{2}Q(E_+) + \frac{1}{2}Q(E_-), \tag{34}$$

$$= -\frac{1}{2\pi}\left[\tan^{-1}\frac{2t_1^2}{\Phi_0^2 - E_+^2} + \tan^{-1}\frac{2t_1^2}{\Phi_0^2 - E_-^2}\right]. \tag{35}$$

Here, $\frac{1}{2}$ is a factor for the total fermion number conservation between zero interfield coupling case ($t_1 = 0$) and nontrivial interfield coupling case ($t_1 \neq 0$). When $t_1 = 0$, the primary mode in the RC soliton system is reduced to the zero mode in the SSH model, which has a fractional fermion number $-\frac{1}{2}$. Hence, using the properties of tangent function, the fermion number in Eq. (34) is modified to

$$Q_{\text{P}}^{\text{RC}} = -\frac{1}{2\pi}\left[\tan^{-1}\frac{2t_1^2}{\Phi_0^2 - E_+^2} + \tan^{-1}\frac{2t_1^2}{\Phi_0^2 - E_-^2}\right] - \frac{1}{2}. \tag{36}$$

Similarly, the fermion number of the induced mode which mainly resides in Ψ_2 can be obtained as below:

$$Q_{\mathrm{I}}^{\mathrm{RC}} = \frac{1}{2\pi}\left[\tan^{-1}\frac{2t_1^2}{\Phi_0^2 - E_+^2} + \tan^{-1}\frac{2t_1^2}{\Phi_0^2 - E_-^2}\right]. \tag{37}$$

Note that the total fermion number of primary and induced modes in an RC soliton system is always quantized as $Q_{\mathrm{P}}^{RC} + Q_{\mathrm{I}}^{RC} = -1/2$ regardless of the strength of t_1.

Using a similar method, the fermion numbers of soliton modes for LC and NC soliton systems can be calculated. The final results are summarized as follows:

$$Q_{\mathrm{P}}^{\mathrm{RC}} = -x - 1/2, \quad Q_{\mathrm{I}}^{\mathrm{RC}} = +x, \tag{38}$$

$$Q_{\mathrm{P}}^{\mathrm{LC}} = +x - 1/2, \quad Q_{\mathrm{I}}^{\mathrm{LC}} = -x, \tag{39}$$

$$Q_{\mathrm{AB}}^{\mathrm{NC}} = -1/2, \qquad Q_{\mathrm{B}}^{\mathrm{NC}} = -1/2, \tag{40}$$

where $x = \frac{1}{2\pi}\left[\tan^{-1}\frac{2t_1^2}{\Phi_0^2 - E_+^2} + \tan^{-1}\frac{2t_1^2}{\Phi_0^2 - E_-^2}\right]$. The opposite signs of x between RC and LC soliton systems indicate that RC and LC solitons form a charge conjugation pair. For an NC soliton system, each fermion number of bonding and antibonding modes is $-1/2$ regardless of t_1 and t_2 values.

S2.4. Comparison with the tight-binding model

We compare the analytically obtained energy spectra and charges of the isolated soliton modes with numerically calculated results at $t_1 = t_2$ case. The numerical calculations were performed using the tight-binding method for the double Peierls chain model [7]. The charge is calculated from the difference of Berry phases between the two global minima because the difference of Berry phases is proportional to the topological charge of a soliton that interpolates the same two global minima[8, 9]. Both results fit well with each other, as shown in Fig. 3(d-i).

S2.5. $\mathcal{T}, \mathcal{C}, \mathcal{P}$ symmetries and dualities among solitons

S2.5.1. Bulk and NC soliton systems

The system has $\mathcal{T}, \mathcal{C}, \mathcal{P}$ symmetries when the bose fields are localized in the AC or BD lines in Fig. 4(c) of the main text. When the bose fields are localized in the AC line, the system has the following symmetries:

$$\mathcal{T}\mathbf{\Psi}(t,x)\mathcal{T}^{-1} = \Gamma_3\mathbf{\Psi}(-t,x), \tag{41}$$

$$\mathcal{C}\mathbf{\Psi}(t,x)\mathcal{C}^{-1} = -\Gamma_{14}\mathbf{\Psi}^*(t,x), \tag{42}$$

$$\mathcal{P}\mathbf{\Psi}(t,x)\mathcal{P}^{-1} = \Gamma_{34}\mathbf{\Psi}(t,-x), \tag{43}$$

$$\mathcal{P}\mathbf{\Phi}(x)\mathcal{P}^{-1} = \mathbf{\Phi}(-x). \tag{44}$$

When the bose fields are in the BD line, the system has the following symmetries:

$$\mathcal{T}\mathbf{\Psi}(t,x)\mathcal{T}^{-1} = \Gamma_3\mathbf{\Psi}(-t,x), \tag{45}$$

$$\mathcal{C}\mathbf{\Psi}(t,x)\mathcal{C}^{-1} = -\Gamma_5\mathbf{\Psi}^*(t,x), \tag{46}$$

$$\mathcal{P}\mathbf{\Psi}(t,x)\mathcal{P}^{-1} = \Gamma_{24}\mathbf{\Psi}(t,-x), \tag{47}$$

$$\mathcal{P}\mathbf{\Phi}(x)\mathcal{P}^{-1} = \mathbf{\Phi}(-x). \tag{48}$$

Hence, the global minima have $\mathcal{T}, \mathcal{C}, \mathcal{P}$ symmetries. Likewise, NC soliton systems that are localized in either AC or BD lines have $\mathcal{T}, \mathcal{C}, \mathcal{P}$ symmetries, which leads that an NC soliton is its own anti-NC soliton.

S2.5.2. RC and LC soliton systems

RC and LC soliton systems have the same $\mathcal{T}$ symmetry:

$$\mathcal{T}\mathbf{\Psi}(t,x)\mathcal{T}^{-1} = \Gamma_3\mathbf{\Psi}(-t,x). \tag{49}$$

On the other hand, $\mathcal{C}$ and $\mathcal{P}$ symmetries are broken. The broken $\mathcal{P}$ symmetry indicates that RC and LC solitons have chiralities.

The charge conjugated and parity transformed fields for an RC soliton system can be described by the help of the field in an LC soliton system and vice versa.

$$\mathcal{C}\mathbf{\Psi}_{\mathrm{RC}(i\to j)}(t,x)\mathcal{C}^{-1} = -\Gamma_{14}U\mathbf{\Psi}^*_{\mathrm{LC}(j\to i)}(t,x), \tag{50}$$

$$\mathcal{P}\mathbf{\Psi}_{\mathrm{RC}(i\to j)}(t,x)\mathcal{P}^{-1} = \Gamma_{34}U^*\mathbf{\Psi}_{\mathrm{LC}(j\to i)}(t,-x), \quad \mathcal{P}\mathbf{\Phi}(x)\mathcal{P}^{-1} = \mathbf{\Phi}(-x), \tag{51}$$

where i and j in the subscript indicate two global minima that the corresponding soliton field connects. And the unitary matrices U are given by

$$U = \begin{pmatrix} 0 & 1 \\ -i\sigma_x & 0 \end{pmatrix}, \text{ when } (i,j) = (A,B) \text{ or } (C,D),$$

$$U = \begin{pmatrix} 0 & i\sigma_x \\ 1 & 0 \end{pmatrix}, \text{ when } (i,j) = (B,C) \text{ or } (D,A).$$

This indicates RC and LC solitons form a particle-antiparticle and chiral pair. One can check such particle-antiparticle duality using equations of motion including $U(1)$ electromagnetic fields. For example, we consider the RC and LC soliton systems with $(i,j) = (A,B)$. Then, the fermion field $\mathbf{\Psi}_{\mathrm{RC}(A\to B)}(t,x)$ satisfies the following equation of motion:

$$[(i\partial_0 - eA_0)\Gamma_2 + (\partial_1 + ieA_1)\Gamma_3 + t_1\Gamma_{34} - it_2\Gamma_{25} + \Phi_s(x)A + \Phi_0 B]\mathbf{\Psi}_{\mathrm{RC}(A\to B)}(t,x) = 0. \tag{52}$$

The charge conjugated field $\mathbf{\Psi}^{\mathcal{C}}_{\mathrm{RC}(A\to B)}(t,x)$ satisfies

$$[(i\partial_0 + eA_0)\Gamma_2 + (\partial_1 - ieA_1)\Gamma_3 + t_1\Gamma_{34} - it_2\Gamma_{25} + \Phi_s(x)A + \Phi_0 B]\mathbf{\Psi}^{\mathcal{C}}_{\mathrm{RC}(A\to B)}(t,x) = 0. \tag{53}$$

The fermion field $\mathbf{\Psi}_{\mathrm{LC}(B\to A)}(t,x)$ in the LC soliton system satisfies

$$[(i\partial_0 - eA_0)\Gamma_2 + (\partial_1 + ieA_1)\Gamma_3 + t_1\Gamma_{34} - it_2\Gamma_{25} - \Phi_s(x)A + \Phi_0 B]\mathbf{\Psi}_{\mathrm{LC}(B\to A)}(t,x) = 0. \tag{54}$$

If we take $\Gamma_{14}U^*$ after applying the complex conjugation to Eq. (54), we get

$$[(i\partial_0 + eA_0)\Gamma_2 + (\partial_1 - ieA_1)\Gamma_3 + t_1\Gamma_{34} - it_2\Gamma_{25} + \Phi_s(x)A + \Phi_0 B](-\Gamma_{14})U\mathbf{\Psi}^*_{\mathrm{LC}(B\to A)}(t,x) = 0. \tag{55}$$

From Eq. (55), one can see that $-\Gamma_{14}U\mathbf{\Psi}^*_{\mathrm{LC}(B\to A)}(t,x)$ satisfies the same equation of motion for the charge conjugated field of an RC soliton system in Eq. (53), which implies Eq. (50). Similarly, the chiral duality between RC and LC solitons can be checked.

S2.6. Field rotation symmetry

Two rotational transformations can be considered in the generalized JR model: π-rotation ($n = 2$) and $\pi/2$-rotation ($n = 1$). The rotation is given by

$$\mathbf{\Psi}(t,x) \to \begin{pmatrix} 0 & -i\sigma_x \\ 1 & 0 \end{pmatrix}^n \mathbf{\Psi}(t,x), \tag{56}$$

$$\mathbf{\Phi}(x) \to e^{i\Gamma_{14}\theta}\mathbf{\Phi}(x), \tag{57}$$

where $e^{i\Gamma_{14}\theta} = \begin{pmatrix} \cos\theta & \sin\theta \\ -\sin\theta & \cos\theta \end{pmatrix}$ and $\theta = \frac{n\pi}{2}$. If we take the $\pi/2$-rotation, t_1 and t_2 are exchanged. Hence, the Lagrangian density is invariant under the $\pi/2$-rotation only when $t_1 = t_2$. Under the π-rotation, t_1 and t_2 exchange twice, which implies that the Lagrangian density is invariant regardless of t_1 and t_2 values.

[1] R. Jackiw and J. Schrieffer, Nuclear Physics B **190**, 253 (1981).
[2] R. Jackiw and C. Rebbi, Phys. Rev. D **13**, 3398 (1976).
[3] W. P. Su, J. R. Schrieffer, and A. J. Heeger, Phys. Rev. Lett. **42**, 1698 (1979).
[4] M. J. Rice and E. J. Mele, Phys. Rev. Lett. **49**, 1455 (1982).
[5] D. Baeriswyl and K. Maki, Phys. Rev. B **28**, 2068 (1983).
[6] J. Goldstone and F. Wilczek, Phys. Rev. Lett. **47**, 986 (1981).
[7] S. Cheon, T.-H. Kim, S.-H. Lee, and H. W. Yeom, Science **350**, 182 (2015).
[8] X.-L. Qi, T. L. Hughes, and S.-C. Zhang, Phys. Rev. B **78**, 195424 (2008).
[9] C.-g. Oh, S.-H. Han, S.-G. Jeong, T.-H. Kim, and S. Cheon, Sci Rep **11**, 1013 (2021).
